# Job crafting and innovative behavior among nurses: The serial mediating effect of team psychological safety and perceived organizational support

**DOI:** 10.1371/journal.pone.0352141

**Published:** 2026-08-10

**Authors:** XuYan Liu, RenLong Liang, Jijun Wu, Yin Yuan, YiWei Li, Tingting Ruan, Heng Ding, Tingting Cai

**Affiliations:** 1 Department of Nursing, Deyang People’s Hospital, Deyang, Sichuan, China; 2 Department of Neurology, Deyang Hospital Affiliated Hospital of Chengdu University of Traditional Chinese Medicine, Deyang, Sichuan, China; 3 Department of Gynecology, Deyang People’s Hospital, Deyang, Sichuan, China; Noida International University, INDIA

## Abstract

Nurses’ innovative behavior plays an important role in improving nursing quality and patient safety. Although job crafting has been reported to be associated with innovative behavior, the underlying mechanisms among nurses remain insufficiently understood. This study aimed to examine the associations among job crafting, team psychological safety, perceived organizational support, and innovative behavior, as well as the individual and serial mediating associations of team psychological safety and perceived organizational support. A cross-sectional correlational study was conducted among 449 nurses from tertiary hospitals in Southwest China. Data were collected using self-reported questionnaires in accordance with the STROBE guidelines. SPSS 27.0 was used for descriptive statistics and Pearson correlation analyses, and Amos 28.0 was used to construct a structural equation model (SEM). Bootstrapping with 5,000 resamples was performed to examine indirect associations. A CFA demonstrated acceptable model fit (χ²/df = 2.822, RMSEA = 0.064, CFI = 0.923, TLI = 0.908, IFI = 0.924). Job crafting was positively associated with innovative behavior (r = 0.473, p < 0.001). Team psychological safety and perceived organizational support both showed significant mediating associations between job crafting and innovative behavior. In addition, team psychological safety and perceived organizational support formed a significant serial mediating association between job crafting and innovative behavior, with bootstrap 95% confidence intervals excluding zero. Job crafting may be associated with nurses’ innovative behavior directly and indirectly through team psychological safety and perceived organizational support. These findings suggest that nursing managers may promote nurses’ innovative participation by encouraging proactive work adjustment, strengthening psychologically safe team climates, and enhancing organizational support resources.

## 1. Introduction

Against the backdrop of refined medical services and diversified nursing needs, nurses’ innovative behavior has become an important driver for improving nursing quality and patient safety. However, nurses’ participation in innovation remains at a moderate level, with rigid work routines and resource scarcity identified as important barriers [1]. Compared with many other occupational groups, nurses often work in highly standardized and risk-sensitive clinical environments, which may increase the difficulty of implementing innovative behaviors. Job crafting, defined as employees’ proactive adjustment of work tasks, interpersonal relationships, and cognitive perceptions of work, has attracted increasing attention as a positive work behavior associated with innovation [2]. Previous studies have shown that job crafting may alleviate nurses’ burnout, enhance work autonomy, and promote innovative activities [3–5]. Team psychological safety and perceived organizational support have also been linked to job crafting and innovative behavior [6].

However, existing evidence regarding the mechanisms underlying the association between job crafting and innovative behavior remains insufficient. Existing studies have largely examined isolated psychological or organizational mechanisms underlying innovative behavior, while the potential sequential interplay between interpersonal psychological resources and broader organizational support perceptions remains insufficiently understood, particularly among nurses.

Therefore, this study focuses on nurses and constructs a serial mediation model to examine the associations among job crafting, team psychological safety, perceived organizational support, and innovative behavior. To our knowledge, few studies have simultaneously examined the serial associations among these variables among nurses. Theoretically, this study may help clarify how interpersonal psychological resources and organizational support resources are jointly associated with nurses’ innovative behavior. Practically, the findings may provide nursing managers with intervention strategies involving behavioral activation, psychological empowerment, and resource support to promote innovative participation.

### 1.1. The relationship between nurses’ job crafting and innovative behavior

According to the Conservation of Resources (COR) theory, job crafting can be viewed as a process through which individuals proactively obtain and optimize work-related resources [7]. Through task, relational, and cognitive crafting, nurses may reduce resource depletion while increasing access to resources such as autonomy, experience, and interpersonal support. These resources may subsequently facilitate innovative behavior [8,9]. For example, nurses may optimize nursing procedures, improve interdisciplinary communication, and reinterpret complex clinical tasks as opportunities for professional development [10–12]. Such proactive adjustments may increase nurses’ willingness and ability to engage in innovative activities [13].

Existing empirical studies have reported positive associations between job crafting and innovative behavior in healthcare settings. Previous studies have reported positive associations between job crafting and innovative behavior among healthcare workers and nurses [2,14,15]. Based on the above theoretical and empirical evidence, we propose the following hypothesis:

*Hypothesis 1: Nurses’ job crafting is significantly positively associated with their innovative behavior.*

### 1.2. The mediating role of team psychological safety

Social Information Processing (SIP) theory suggests that individuals’ psychological perceptions are influenced by social information in the work environment [16]. Nurses engaging in job crafting may improve communication patterns and collaborative interactions within teams, thereby increasing perceptions of psychological safety [17,18]. Team psychological safety refers to a shared belief that individuals can express ideas and concerns without fear of negative interpersonal consequences. Previous studies have shown that psychologically safe environments may reduce concerns about mistakes and encourage idea sharing and innovative participation [19–21].

Existing studies also indirectly support the mediating role of team psychological safety. A cross-sectional study of nurses reported a positive correlation between team psychological safety and job crafting [22], while Elsayed et al. [23] found that team psychological safety was positively associated with employees’ innovative work behavior. Based on the above theory and empirical evidence, we propose:

*Hypothesis 2: Team psychological safety may statistically mediate the association between nurses’ job crafting and innovative behavior.*

### 1.3. The mediating role of perceived organizational support

Social Exchange Theory (SET) suggests that employees who perceive organizational recognition and support may be more willing to engage in positive work behaviors [24]. Nurses’ engagement in job crafting may reflect proactive work contributions, which may subsequently enhance organizational recognition and support [25]. Perceived organizational support reflects employees’ perceptions that the organization values their contributions and cares about their well-being.

Perceived organizational support has also been reported to be positively associated with innovative behavior. Organizational support may provide nurses with training opportunities, emotional encouragement, and innovation-related resources, thereby reducing perceived constraints during innovation processes [26]. Previous studies have reported positive associations among perceived organizational support, job crafting, and innovative behavior [27]. Based on the above theory and empirical evidence, we propose:

*Hypothesis 3: Perceived organizational support may statistically mediate the association between nurses’ job crafting and innovative behavior.*

### 1.4. The serial mediating association of team psychological safety and perceived organizational support

Team psychological safety and perceived organizational support may further form a serial mediating association between job crafting and innovative behavior. Although both variables represent positive workplace resources, team psychological safety mainly reflects an interpersonal psychological climate within the team, whereas perceived organizational support reflects broader organizational recognition and resource support.

According to SIP theory [16], nurses with higher team psychological safety may be more willing to communicate openly, express work-related needs, and actively participate in organizational affairs. Such proactive engagement may subsequently enhance perceptions of organizational support. Therefore, team psychological safety may function as a proximal interpersonal resource that may be related to higher levels of perceived organizational support as a broader organizational resource.

Compared with previous studies examining team psychological safety or perceived organizational support separately, the present study further explores their potential sequential associations within a unified framework among nurses. Thus, we propose:

*Hypothesis 4: Team psychological safety and perceived organizational support may statistically form a serial mediating association between nurses’ job crafting and innovative behavior.*

**Fig 1:**
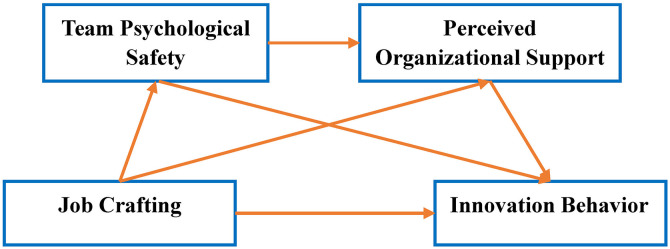
Hypothesized serial mediation model.

Integrating COR, SIP, and SET provides a coherent framework for understanding the sequential mechanism proposed in this study. From the COR perspective, job crafting enables nurses to proactively acquire and optimize work-related resources. SIP theory further suggests that these proactive experiences may enhance psychological safety through positive interpersonal cues within team interactions. Building on this psychological foundation, SET proposes that nurses who perceive stronger organizational support may feel greater obligation and motivation to reciprocate through proactive innovative behavior. The hypothesized serial mediation model is presented in Fig 1.

## 2. Materials and methods

### 2.1. Study design

This study adopted a descriptive, cross-sectional correlational study design and adhered to the STROBE reporting guidelines.

### 2.2. Sample size calculation

The sample size was calculated using G*Power 3.1 software. According to Cohen’s criteria [28], the significance level was set at 0.05, the statistical power at 95%, and a medium effect size was specified with an f² value of 0.15. The total number of predictor variables in this study was 15. Based on these parameters, G*Power calculated the required sample size as 204 cases. To obtain more reliable results, we intended to include as many samples as possible under feasible conditions, and finally planned to invite 500 nurses to participate in the survey.

### 2.3. Study participants

A convenience sampling method was used to recruit 500 nurses from three Grade A tertiary general hospitals in western China. Seventeen respondents withdrew from the study midway. Fourteen questionnaires with incomplete responses and 20 with unreasonable responses were excluded.Finally, a total of 449 questionnaires were included in the analysis, with an effective response rate of 89.80%.

The inclusion criteria of this study were as follows: Being an in-service registered nurse; Working in the clinical frontline; Having work experience of ≥ 1 year; Providing informed consent and voluntarily cooperating to complete the survey.

The exclusion criteria of this study were as follows: Being absent from work due to external study, training, sick leave, personal leave, maternity leave, or other reasons; Being student nurses or visiting nurses.

### 2.4. Ethical considerations

This study strictly adhered to the medical ethics guidelines stipulated in the Helsinki Declaration and was reviewed and approved by the Ethics Committee of Deyang People’s Hospital (Approval No.: 2024-04-016-K01). An informed consent form was placed prior to the questionnaire content, through which participants were fully informed of the study purpose, implementation procedures, and potential relevant risks. The subsequent survey was conducted only after obtaining explicit informed consent from the participants. In addition, participants’ personal information falls within the scope of protected privacy and confidentiality. During the entire research process and the subsequent data collation stage, this information will be anonymized, and sensitive information such as participants’ phone numbers and addresses will not be collected, so as to effectively safeguard participants’ information security and legitimate rights and interests.

### 2.5. Research tools

#### 2.5.1. General information survey form.

The General Information Questionnaire was designed based on the results of previous literature studies. The questionnaire covers the following items: Gender; Age; Educational level; Marital status; Professional title; Length of service; Whether they are night-shift nurses; Whether they have participated in innovation training; The size of their nursing team; Whether innovation rewards are provided; Department.

#### 2.5.2. Job Crafting Scale.

The Job Crafting Scale, developed by Dvorak [29] and adapted into Chinese by Zhu et al. [30], was used in this study. This scale consists of 3 dimensions and a total of 21 items, including: Task Crafting (7 items); Cognitive Crafting (7 items); Relational Crafting (7 items). It uses a 5-point Likert scale, with scores ranging from 1 to 5. The scores correspond to “Strongly Disagree” (1 point) to “Strongly Agree” (5 points).The total score range is 21–105 points. A higher score indicates more job crafting behaviors among nurses. The overall Cronbach’s α coefficient of the original scale was 0.920 [30], and the Cronbach’s α coefficient of this scale in the current study was 0.893.

#### 2.5.3. Team Psychological Safety Scale.

The Team Psychological Safety Scale, developed by Edmondson [31], consists of a single dimension with 7 items. It uses a 6-point Likert scale, with scores ranging from 1 to 6. The scores correspond to “Strongly Disagree” (1 point) to “Strongly Agree” (6 points). The total score of the scale ranges from 7 to 42. A higher score indicates a higher level of Team Psychological Safety. The Cronbach’s α coefficient of the scale was 0.802 [31], and the Cronbach’s α coefficient measured in this study was 0.728.

#### 2.5.4. Perceived Organizational Support Scale.

The Perceived Organizational Support Scale was first developed by Eisenberger et al. [32].Zuo et al. [33] adapted and revised it to form the Chinese version of the Perceived Organizational Support Scale. This scale consists of 2 dimensions and a total of 13 items, including: Emotional Support (10 items); Instrumental Support (3 items). It uses a 5-point Likert scale, with scores ranging from 1 to 5 corresponding to “Strongly Disagree” (1 point) to “Strongly Agree” (5 points).The total score range is 13–65 points. A higher score indicates a higher level of Perceived Organizational Support among nurses. The Cronbach’s α coefficient of the scale was 0.870 [33], and the Cronbach’s α coefficient of this scale in the current study was 0.842.

#### 2.5.5. Innovative Behavior Scale.

Lukes et al. [34] developed the Innovative Behavior Inventory (IBI).Huang et al. [35] adapted it into Chinese in line with Chinese cultural characteristics. After translation, back-translation, and cultural adaptation, 3 items were deleted from the scale. It was finally confirmed to contain 20 items, with 5 dimensions as follows: Idea Generation and Search (6 items); Plan Communication and Implementation (5 items); Acquisition of Human Resources (3 items); Overcoming Barriers (3 items); Clinical Application (3 items). It uses a 5-point Likert scale, with scores ranging from 1 to 5. The scores correspond to “Strongly Disagree” (1 point) to “Strongly Agree” (5 points).The total score ranges from 20 to 100 points. A higher score indicates stronger innovative behavior ability among nurses. The Cronbach’s α coefficient of the total scale was 0.923, and the test-retest reliability was 0.952, showing good reliability and validity [35].The Cronbach’s α coefficient of this scale in the current study was 0.842.

### 2.6. Data collection and quality control methods

From June 8, 2025 to July 28, 2025, with the approval of the hospitals where the respondents were employed, the researchers completed the questionnaire distribution with their assistance. This survey was conducted via the WeChat-based platform “Wenjuanxing”. Detailed explanations of the study’s purpose, significance, core content, and inclusion/exclusion criteria were provided in the questionnaire, and it was emphasized that respondents should answer carefully and truthfully based on their actual situations. Meanwhile, the respondents were clearly informed of the following matters: their personal information would be strictly kept confidential and used solely for research data analysis; participation in the survey was voluntary; and the entire response process was anonymous. Completion guidelines were attached to the first page of the questionnaire and each module. The system was configured such that each IP address or WeChat account could only submit the questionnaire once, with a submission time limit of 8 minutes. Based on the results of the pilot survey and the average completion time required to read and answer all questionnaire items carefully, responses completed in less than 8 minutes were considered potentially invalid due to insufficient response time. After questionnaire collection, two researchers jointly conducted a thorough review of the questionnaires and entered the data, simultaneously excluding invalid questionnaires with logically contradictory answers, obvious errors, or patterned response behaviors. Any discrepancies arising during the review process were resolved through joint discussions among the research team members.

### 2.7. Statistical analyses

Data analysis was performed using SPSS 27.0. Demographic characteristics of participants were described with frequencies and percentages. After conducting normality tests on all scale items, the scale scores were presented as mean ± standard deviation (SD). Pearson correlation analysis was used to examine the correlations among variables. Harman’s single-factor analysis of variance (ANOVA) was employed to detect common method bias. Prior to SEM analysis, multicollinearity among the study variables was assessed using variance inflation factor (VIF) statistics.SEM was constructed using IBM SPSS Amos 28 Graphics.CFA was conducted using Amos 28.0 to evaluate the measurement model before testing the structural relationships among variables. Composite reliability (CR) and average variance extracted (AVE) were calculated to further assess construct reliability and convergent validity. Mediation effects were tested via the Bootstrapping method (n = 5,000), with CIs calculated. A mediation effect was considered statistically significant if the 95% CI did not include zero, and the significance level was set at α = 0.05 [36].

## 3. Results

### 3.1. Common method bias test

Several procedural remedies were adopted to reduce common method bias, including anonymous participation, voluntary responses, confidentiality assurances, and standardized questionnaire instructions. Harman’s single-factor ANOVA was used to test for common method bias across all variables. The results showed that all 15 factors had eigenvalues greater than 1, and the variance explained by the first factor was 21.90%—which is below the critical value of 40%. These findings suggest that common method bias may not seriously threaten the study results. However, Harman’s single-factor test has recognized limitations and cannot completely rule out the potential influence of common method variance(CMV). The VIF values ranged from 1.341 to 1.397, all below the recommended threshold of 5, indicating that multicollinearity was not a serious concern.

### 3.2. General information of the surveyed nurses

A total of 449 valid questionnaires were finally included in this study for analysis. The results showed that gender, marital status, length of service, and department had no significant effect on nurses’ innovative behavior.While age, educational level, professional title, whether they are night-shift nurses, whether they have participated in innovation training, the size of their nursing team, and whether innovation rewards are provided had a significant effect on nurses’ innovative behavior. The general demographic information of the surveyed nurses is shown in Table 1.

**Table 1:** General information of interviewees and scores of innovative behavior under different variables.

Variables	n(%)	x̅ ± s	*F*	*p*
Gender				
Male	91(20.27%)	67.66 ± 12.75	0.432	0.511
Female	358(79.73%)	68.56 ± 11.45		
Age				
Aged 20–30	174(38.75%)	66.90 ± 11.85	8.116	<0.001
Aged 31–40	209(46.55%)	70.65 ± 10.99		
Aged 41 and above	66(14.70%)	65.11 ± 12.37		
Marital status				
Not married	119(26.50%)	68.40 ± 11.23	0.541	0.583
Married	304(67.71%)	68.57 ± 11.88		
Divorced	26(5.79%)	66.08 ± 12.26		
Education level				
Junior college	89(19.82%)	61.88 ± 13.15	24.067	<0.001
Bachelor’s	325(72.38%)	69.37 ± 10.35		
Master’s	35(7.80%)	75.77 ± 12.86		
Professional title				
Junior nurse	225(50.11%)	68.04 ± 11.87	3.650	0.027
Intermediate nurse	183(40.76%)	67.76 ± 11.97		
Senior nurse	41(9.13%)	73.05 ± 8.500		
Length of service				
1-5 years	127(28.29%)	69.03 ± 10.13	1.039	0.375
6-10 years	131(29.18%)	66.88 ± 13.03		
11-20 years	127(28.29%)	68.82 ± 12.44		
>20 years	64(14.25%)	69.30 ± 10.24		
Whether they are night-shift nurses				
YES	272(60.58%)	64.91 ± 11.07	69.748	<0.001
NO	177(39.42%)	73.71 ± 10.66		
Whether the Nurse Has Participated in Innovation Training				
YES	237(52.78%)	71.70 ± 10.97	44.284	<0.001
NO	212(47.22%)	64.67 ± 11.43		
The size of their nursing team				
≤3	117(26.06%)	62.09 ± 12.73	28.993	<0.001
4-6	249(55.46%)	69.09 ± 10.40		
≥7	83(18.49%)	73.33 ± 10.38		
Whether innovation rewards are provided				
YES	383(85.30%)	69.75 ± 11.24	38.351	<0.001
NO	66(14.70%)	60.45 ± 11.34		
Department				
Internal medicine	133(29.62%)	68.93 ± 11.41	0.564	0.759
Surgery	105(23.39%)	67.17 ± 12.20		
Outpatient and emergency department	39(8.69%)	68.64 ± 10.19		
Intensive care unit	33(7.35%)	67.03 ± 11.18		
Obstetrics, Gynecology and Pediatrics	37(8.24%)	67.38 ± 15.35		
Operating room	31(6.90%)	68.94 ± 10.88		
Other	71(15.81%)	69.90 ± 10.97		

### 3.3. Correlation analysis between variables

The scores of each variable were described using mean ± standard deviation (SD), and Pearson correlation analysis was conducted to examine the relationships among the study variables. The results showed that job crafting was significantly positively correlated with innovative behavior (r = 0.473, p < 0.001), team psychological safety (r = 0.426, p < 0.001), and perceived organizational support (r = 0.463, p < 0.001). In addition, team psychological safety was significantly positively correlated with innovative behavior (r = 0.462, p < 0.001) and perceived organizational support (r = 0.436, p < 0.001), while perceived organizational support was significantly positively correlated with innovative behavior (r = 0.513, p < 0.001). These findings provided preliminary support for the hypothesized relationships among the variables. Detailed results are presented in Table 2.

**Table 2:** Correlations Among Variables.

Variables	Job Crafting	Perceived Organizational Support	Team Psychological Safety	Innovative Behavior
Job Crafting	1	–	–	–
Perceived Organizational Support	0.463^**^	1	–	–
Team Psychological Safety	0.426^**^	0.436^**^	1	–
Innovative Behavior	0.473^**^	0.513^**^	0.462^**^	1
M ± SD	67.55 ± 12.80	41.97 ± 7.53	30.29 ± 4.52	68.38 ± 11.72

** P < 0.001.

### 3.4. Measurement model assessment

A CFA was conducted to evaluate the measurement model. The results demonstrated an acceptable model fit: χ²/df = 2.822, RMSEA = 0.064, CFI = 0.923, TLI = 0.908, and IFI = 0.924, indicating satisfactory construct validity of the measurement model.See Table 3 for details.

**Table 3:** SEM fitting index.

Project	Statistical tests	Suggested reference standards	Test results
Absolute fit indices	χ²/df	≤3	2.822
	RMSEA	≤0.08	0.064
Relative fit indices	CFI	≥0.90	0.923
	TLI	≥0.90	0.908
	IFI	≥0.90	0.924
Parsimony fit index	PCFI	The closer to 1, the better.	0.767
	PNFI	The closer to 1, the better.	0.737

RMSEA:Root Mean Square Error of Approximation, CFI:Comparative Fit Index, TLI:Tucker-Lewis Index, IFI:Incremental Fit Index, PCFI:Parsimonious Comparative Fit Index, PNFI:Parsimonious Normed Fit Index.

The reliability and convergent validity of the latent constructs were further evaluated using Cronbach’s α, CR, and AVE. The Cronbach’s α and CR values of all constructs exceeded the recommended threshold of 0.70, indicating acceptable internal consistency reliability. See Table 4 for details.Although the AVE value of the Team Psychological Safety construct was below the recommended threshold of 0.50, its CR exceeded 0.70, suggesting acceptable convergent validity according to the criterion proposed by Fornell and Larcker.

**Table 4:** Reliability and validity analysis of latent constructs.

Construct	Standardized loading range	Cronbach’s α	CR	AVE
Job Crafting	0.752–0.869	0.879	0.864	0.679
Team Psychological Safety	0.438–0.650	0.728	0.736	0.287
Perceived Organizational Support	0.699–0.874	0.842	0.768	0.626
Innovative Behavior	0.621–0.764	0.903	0.828	0.493

CR:composite reliability; AVE:average variance extracted.

### 3.5. Analysis of serial mediating effects between variables

**Fig 2:**
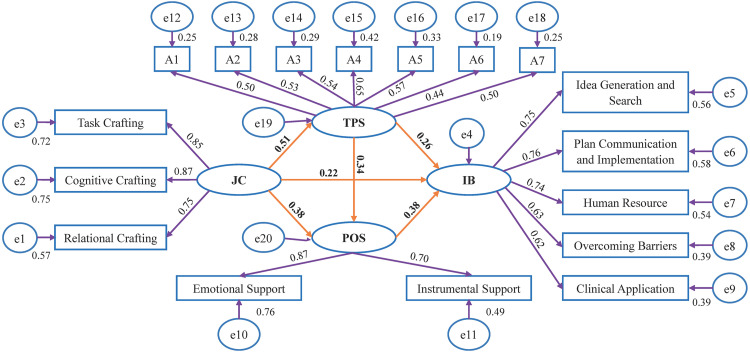
Structural equation model with standardized path coefficients. Note: JC:Job Crafting; TPS:Team Psychological Safety; POS:Perceived Organizational Support; IB: Innovative Behavior;A1-A8:TPS items.

Amos 28 software was used to construct a SEM. In this model, nurses’ job crafting was the independent variable, Team Psychological Safety and Perceived Organizational Support were the mediating variables, and innovative behavior was the dependent variable. The maximum likelihood method was used to fit and modify the model. The final model was established, as shown in Fig 2. Model fitting results are shown in Table 4. All parameters were within the ideal range, indicating a good model fit.

Bootstrapping with 5,000 resamples was performed to examine the mediating associations. A 95% bootstrap confidence interval (95% CI) excluding zero was considered indicative of a statistically significant effect. The results showed that the indirect association through team psychological safety was significant (β = 0.133, 95% CI [0.064, 0.241]), accounting for 23.71% of the total effect. The indirect association through perceived organizational support was also significant (β = 0.143, 95% CI [0.044, 0.253]), accounting for 25.31% of the total effect. In addition, the serial indirect association through team psychological safety and perceived organizational support was significant (β = 0.067, 95% CI [0.023, 0.137]). Because none of the bootstrap confidence intervals included zero, all indirect associations were considered statistically significant. Therefore, Hypotheses 2–4 were supported. Detailed results are presented in Table 5.

**Table 5:** Analysis Results of the Mediating Effect of Team Psychological Safety and Perceived Organizational Support.

Type of Effect	Effect Size	Boot SE	95% CI Lower	95% CI Upper	P	Percentage
Total Effect	0.561	0.039	0.494	0.621	0.001	100%
Direct Effect	0.219	0.076	0.091	0.338	0.006	39.04%
Total Indirect Effect	0.342	0.070	0.234	0.461	<0.001	60.96%
X → M1 → Y	0.133	0.064	0.041	0.247	0.011	23.71%
X → M2 → Y	0.143	0.044	0.080	0.226	<0.001	25.31%
X → M1 → M2 → Y	0.067	0.023	0.037	0.113	<0.001	11.94%

X: Job Crafting;M1: Team Psychological Safety;M2: Perceived Organizational Support;Y: Innovative Behavior.

## 4. Discussion

### 4.1. General status of each variable

In this study, the mean score for nurses’ job crafting was 67.55 ± 12.80, which was slightly higher than the scores reported by Yang et al. [37] and Zhao et al. [38]. One possible explanation may be related to differences in clinical work settings. Previous studies mainly focused on emergency department and ICU nurses, whose heavier workloads and higher work intensity may limit opportunities for proactive work adjustment and job crafting behaviors.

The mean score for team psychological safety was 30.29 ± 4.52, which was higher than that reported by Pfeifer et al. [39] among American pediatric nurses. This difference may partly be related to differences in clinical environments and work characteristics. Pediatric nurses often face relatively high interpersonal and operational pressures due to concerns about disputes arising from clinical errors, which may reduce perceptions of psychological safety. In addition, some studies have suggested that collectivist-oriented workplace environments may facilitate interpersonal coordination and team cohesion. However, because cultural variables were not directly measured in the present study, such interpretations should be treated cautiously and require further empirical verification.

The mean score for perceived organizational support was 41.97 ± 7.53, which was generally consistent with the findings reported by Ren et al. [40] and Liu et al. [41]. This finding suggests that Chinese nurses generally perceive a moderate level of organizational support. Similarly, the mean score for innovative behavior was 68.38 ± 11.72, which was close to the result reported by Liu et al. [42] but lower than that reported by Wang et al. [43]. Differences across studies may be related to variations in institutional innovation climates, research training opportunities, and hospital management systems.

Overall, all variables were at above-average levels, indicating that nurses generally demonstrated relatively positive levels of proactive work adjustment, team psychological safety, perceived organizational support, and innovative behavior. These findings may reflect ongoing improvements in nursing management and organizational support systems in recent years.

### 4.2. The independent mediating associations of team psychological safety and perceived organizational support

This study supported Hypotheses 1–3. Specifically, job crafting was positively associated with team psychological safety and perceived organizational support, and both variables independently mediated the association between job crafting and innovative behavior. The indirect association through team psychological safety accounted for 23.71% of the total effect, while the indirect association through perceived organizational support accounted for 25.31% of the total effect. The bootstrap confidence intervals for both indirect associations did not include zero, indicating statistically significant mediating associations.

These findings are generally consistent with the COR theory, which suggests that individuals may improve their ability to cope with work challenges by actively acquiring and maintaining resources [44]. In the present study, nurses who engaged in job crafting may have accumulated psychological and organizational resources through proactive work adjustment. For example, task crafting may enhance work autonomy, whereas relational crafting may strengthen interpersonal support within the team. These resources may help reduce concerns about innovation-related uncertainty and facilitate greater participation in innovative activities [45].

The findings also suggest that team psychological safety and perceived organizational support may play different but complementary roles in the association between job crafting and innovative behavior. Team psychological safety mainly reflects the interpersonal climate within the team and may encourage nurses to express ideas and participate in innovation without excessive fear of negative evaluation. In contrast, perceived organizational support primarily reflects broader institutional recognition and resource support, such as training opportunities, managerial encouragement, and innovation-related resources. Consistent with previous nursing and organizational studies [25,46], nurses who proactively adjust their work may also be more likely to receive recognition and support from their organizations.

From a practical perspective, the findings suggest that nursing managers should not only encourage proactive work adjustment among nurses but also strengthen psychologically safe team climates and organizational support systems simultaneously. Interventions targeting only one aspect may be insufficient to fully promote innovative behavior.

### 4.3. The serial mediating association of team psychological safety and perceived organizational support

This study further found that job crafting was indirectly associated with innovative behavior through the serial pathway of “team psychological safety → perceived organizational support,” supporting Hypothesis 4. The bootstrap confidence interval for the serial indirect association did not include zero, indicating a statistically significant serial mediating association.

Compared with previous studies that examined team psychological safety or perceived organizational support separately, the present study further suggests that these two variables may be statistically interrelated within a unified framework. Team psychological safety and perceived organizational support, although related, represent different levels of workplace resources. Team psychological safety mainly reflects interpersonal psychological resources within teams, whereas perceived organizational support reflects broader organizational-level resource perceptions.

According to SIP [16], nurses who perceive higher psychological safety may be more willing to communicate openly, express work-related needs, and actively participate in organizational affairs. Such proactive engagement may help strengthen perceptions of organizational support. In addition, previous studies have suggested that psychologically safe environments may facilitate communication, information sharing, and organizational participation [47,48], which may further enhance perceptions of support from the organization.

The present findings extend previous research by suggesting a potential serial association pathway linking psychological and organizational resources with innovative behavior. However, because this study adopted a cross-sectional design, the temporal ordering of these variables should be interpreted cautiously, and alternative explanations remain possible. Future longitudinal or experimental studies are needed to further verify the causal and temporal relationships among these variables.

From a practical perspective, the findings suggest that nursing managers may benefit from developing integrated intervention strategies rather than focusing on isolated factors. For example, managers may encourage nurses’ proactive work adjustment while simultaneously fostering psychologically safe team environments and providing stronger organizational support for innovation-related activities. Such multilevel interventions may help promote nurses’ innovative participation more effectively.

## 5. Conclusion

Job crafting has a significant direct positive association on nurses’ innovative behavior. Team Psychological Safety and Perceived Organizational Support respectively play significant mediating roles between job crafting and nurses’ innovative behavior.Meanwhile, Team Psychological Safety and Perceived Organizational Support play a serial mediating role in the effect of job crafting on nurses’ innovative behavior. Nursing managers should formulate strategies from three dimensions: behavior activation, psychological empowerment, and resource guarantee.These strategies aim to promote the transformation of nursing innovative behavior from passive compliance to active innovation, and facilitate the improvement of nursing quality and nurses’ professional development.

## 6. Practical implications

The findings of this study provide several practical implications for nursing management. First, nursing managers may promote nurses’ innovative engagement through behavioral activation strategies. For example, hospitals may encourage nurses to participate in interdisciplinary quality-improvement projects, nursing innovation competitions, and flexible task redesign initiatives. Providing opportunities for nurses to proactively optimize work processes may help strengthen job crafting and stimulate innovative participation.

Second, psychological empowerment strategies may help strengthen nurses’ team psychological safety. Nursing managers may establish non-punitive communication mechanisms, reflective debriefing meetings, and supportive feedback systems to encourage nurses to openly express ideas and concerns without fear of negative evaluation. Such psychologically safe environments may facilitate knowledge sharing and innovative behaviors.

Third, organizations may strengthen resource guarantee mechanisms to enhance nurses’ perceived organizational support. Hospitals may provide innovation-related training programs, protected innovation time, managerial recognition systems, and financial support for nursing improvement projects. These organizational resources may reduce perceived barriers to innovation and reinforce nurses’ willingness to engage in innovative activities.

Together, these multidimensional interventions may help hospitals establish a supportive innovation climate that promotes nurses’ proactive participation in nursing innovation and quality improvement.

## 7. Study limitations

This study has several limitations. First, all variables were collected through self-reported questionnaires at a single time point, which may increase the risk of CMV. Although Harman’s single-factor test suggested that common method bias was not severe, this approach has recognized methodological limitations and cannot completely exclude potential CMV effects. Future studies are encouraged to adopt more rigorous approaches, such as longitudinal designs, multi-source data collection, or latent method factor techniques, to further reduce common method bias.

Second, this study adopted a cross-sectional design, which limits the ability to infer causal or temporal relationships among variables. Although the mediation model demonstrated statistically significant indirect associations, these findings should not be interpreted as evidence of causality or directional effects. Future longitudinal or experimental studies are needed to further verify the temporal ordering and potential causal mechanisms among job crafting, team psychological safety, perceived organizational support, and innovative behavior.

Third, the study participants were recruited from Southwest China. Due to regional differences in economic development and healthcare environments, the findings may not be fully generalizable to other regions, particularly economically developed coastal areas. Future studies are encouraged to conduct multi-regional and large-sample investigations to improve the generalizability of the findings.

Finally, although several demographic variables were significantly associated with innovative behavior in the preliminary analyses, these variables were not included as covariates in the final SEM model. Future studies should consider controlling for relevant demographic factors to further reduce potential confounding effects and improve the robustness of the findings.

## Supporting information

S1 File Raw data. (XLSX)
