# Supplementary material for: Job crafting and innovative behavior among nurses: The serial mediating effect of team psychological safety and perceived organizational support
Source: PLoS One. 2026 Aug 10;21(8):e0352141. doi: 10.1371/journal.pone.0352141 (PMC13456283; doi:10.1371/journal.pone.0352141)
Supplement: Supplementary file 1 [file pone.0352141.s003.doc]

**Dear Editor and Reviewers,**

**We sincerely thank the Editor and Reviewers for the careful evaluation of our manuscript and for the constructive comments and valuable suggestions. We have carefully revised the manuscript according to all comments and believe that these revisions have substantially improved the clarity, rigor, and overall quality of the manuscript. All changes have been highlighted in the revised version, and detailed responses to each comment are provided below.**

**Response to Reviewer 1,**

**Question 1**

3.1 Causality Claims in Cross-Sectional Design

The manuscript repeatedly uses causal language:

“predictive effect”

“causal relationships”

“transmission chain”

However, the study is cross-sectional. Causality cannot be inferred.

Recommendation:

Replace causal terminology with associative language (e.g., “associated with,” “related to”).

Revise Section 3.4 where the authors state they “verify causal relationships.”

Add a clearer limitation in Section 7 explicitly stating that mediation in cross-sectional data does not imply temporal causation.

Thank you very much for this insightful comment. We fully agree that causal inferences cannot be established based on a cross-sectional design. Following your suggestion, we carefully revised the manuscript to avoid causal language throughout the text.

Specifically, terms such as “predictive effect,” “causal relationship,” and “transmission chain” were replaced with more appropriate associative expressions, including “association,” “relationship,” and “serial mediating association.” In Section 3.4, the statement regarding “verification of causal relationships” has been revised to reflect statistical associations rather than causality. In addition, we strengthened the limitations section by explicitly stating that mediation effects identified from cross-sectional data do not imply temporal or causal relationships among variables.

These revisions have been incorporated throughout the manuscript to ensure conceptual and methodological accuracy.

**Question 2**

3.2 Common Method Bias Assessment Is Insufficient

Harman’s single-factor test is widely criticized as inadequate.

Given:

All data are self-reported

Same-source measurement

Single time point

Risk of common method variance (CMV) is high.

Recommendation:

Conduct additional tests (e.g., CFA marker variable technique or unmeasured latent method factor).

At minimum, acknowledge Harman’s test limitations in the Discussion.

Following your suggestion, we have revised the manuscript in several ways. First, we moderated the interpretation of the Harman’s single-factor test results in Section 3.1. Specifically, instead of stating that “there is no significant common method bias,” we now indicate that the findings only suggest that CMV may not seriously threaten the study results, while acknowledging the recognized limitations of Harman’s test.

Second, we added a clearer discussion in the limitations section to acknowledge the potential risk of CMV associated with the cross-sectional self-report design. We also noted that Harman’s single-factor test cannot completely rule out CMV effects and suggested that future studies adopt more rigorous approaches, such as longitudinal designs, multi-source data collection, or latent method factor techniques.

In addition, we clarified the procedural remedies used to reduce CMV risk, including anonymous participation, voluntary responses, confidentiality assurances, and standardized questionnaire instructions.

These revisions have been incorporated into the revised manuscript accordingly.

**Question 3**

3.3 Convenience Sampling + “Random Recruitment” Inconsistency

Section 2.3 states:

“Convenience sampling method was used”

“500 nurses were randomly recruited”

This is contradictory.

Recommendation:

Clarify:

Was it convenience sampling at hospital level and random within hospitals?

Or purely convenience sampling?

Revise wording for methodological transparency.

Thanks. We agree that the description of the sampling procedure in the original manuscript was unclear and could lead to confusion between convenience sampling and random sampling.

In the revised manuscript, we clarified the sampling strategy by removing the term “randomly recruited.” The study actually adopted a convenience sampling approach, in which eligible nurses from three Grade A tertiary general hospitals in western China were invited to voluntarily participate in the survey.

The relevant description in Section 2.3 has been revised accordingly to improve methodological transparency and accuracy.

**Question 4**

3.4 Reliability of Team Psychological Safety Scale

Cronbach’s α = 0.728 in this study.

While acceptable, it is relatively low compared to original scale.

Recommendation:

Report item-total correlations.

Consider reporting composite reliability (CR) and AVE for SEM constructs.

Clarify whether CFA was conducted for measurement model.

Thank you very much for this constructive comment. Following your suggestion, we further evaluated the measurement properties of the constructs by conducting CFA and calculating CR and AVE.

The CFA results demonstrated an acceptable model fit (χ²/df = 2.822, RMSEA = 0.064, CFI = 0.923, TLI = 0.908, IFI = 0.924). For the Team Psychological Safety Scale, the Cronbach’s α coefficient was 0.728 and the CR value was 0.736, indicating acceptable internal consistency reliability. Although the AVE value was below the recommended threshold of 0.50, the CR exceeded 0.70, which still suggests acceptable convergent validity according to the criterion proposed by Fornell and Larcker.

In addition, item-total correlations and measurement model information have been supplemented in the revised manuscript to improve methodological transparency and rigor.

**Question 5**

3.5 Data Availability Statement Inconsistency

In the submission form:

“All relevant data are within the manuscript and its Supporting Information files.”

But in the manuscript:

“The raw data… will be made available by the authors…”

This is inconsistent with PLOS data policy.

Recommendation:

Ensure full compliance.

If data are not in repository, deposit in public repository before publication.

Revise manuscript to match submission declaration.

Thank you very much for pointing out the inconsistency in the Data Availability Statement.

Following the journal’s data-sharing policy and your suggestion, we have revised the manuscript to ensure full compliance with PLOS data availability requirements. Specifically, the de-identified raw dataset has now been deposited in a public repository (Figshare), and the corresponding DOI link has been added to the revised manuscript.

The previous statement indicating that the data would be available “upon reasonable request” has been removed to avoid inconsistency with the submission information.

The revised Data Availability Statement is as follows:

“The de-identified datasets generated and analyzed during the current study are publicly available in the Figshare repository: https://doi.org/10.6084/m9.figshare.32286552.”

**Question 6**

3.6 Hypothesis Numbering Errors

In Section 3.3:

Hypothesis 5 and Hypothesis 6 are mentioned

Earlier only 4 hypotheses are defined

This reflects structural inconsistency.

Recommendation:

Revise hypothesis numbering.

Ensure consistency between Introduction and Results.

Thank you very much for this careful observation. We apologize for the inconsistency in the hypothesis numbering between the Introduction and Results sections.

Following your suggestion, we carefully checked and revised the hypothesis numbering throughout the manuscript to ensure consistency and structural accuracy. The incorrect references to “Hypothesis 5” and “Hypothesis 6” in Section 3.3 have been corrected to match the four hypotheses originally proposed in the Introduction.

All related sections, tables, and figure descriptions have also been reviewed and revised accordingly.

**Question 7**

4. Methodological Comments

4.1 Control Variables

Demographic variables significantly related to innovative behavior were identified (e.g., age, education, training).

However:

It is unclear whether these were controlled in SEM.

Recommendation:

Include significant demographics as covariates in the SEM model.

Clarify whether this was done.

Thanks. We agree that demographic variables significantly associated with innovative behavior may potentially influence the relationships examined in the SEM model.

In the present study, the primary focus was on the associations among job crafting, team psychological safety, perceived organizational support, and innovative behavior. Demographic variables were examined in the preliminary analyses; however, they were not included as covariates in the final SEM model. We acknowledge that this may limit the ability to fully account for potential confounding effects.

Following your suggestion, we have clarified this issue in the revised manuscript and added it to the limitations section.

**Question 8**

4.2 Stepwise Regression

The manuscript uses stepwise regression.

Stepwise methods are statistically unstable and often discouraged.

Recommendation:

Replace with hierarchical regression based on theory.

Or remove regression section if SEM is primary analysis.

Thanks. We agree that stepwise regression methods may have limitations in terms of statistical stability and theoretical interpretability.

Considering that structural equation modeling (SEM) was the primary analytical approach of this study, we carefully reconsidered the necessity of the stepwise regression analysis. Following your suggestion, we removed the stepwise regression results from the revised manuscript and retained SEM as the main analytical framework for examining the relationships among variables.

The manuscript has been revised accordingly to improve methodological rigor and consistency.

**Question 9**

4.3 VIF Reporting

You report VIF < 5.

Provide:

Exact VIF range.

Clarify whether multicollinearity was tested before SEM.

Thank you very much for this helpful suggestion. We agree that more detailed reporting of multicollinearity diagnostics would improve the transparency of the analysis.

Following your recommendation, we clarified in the revised manuscript that multicollinearity diagnostics were conducted prior to the SEM analysis. Specifically, VIF values were calculated to assess potential multicollinearity among the study variables.

In addition, we supplemented the manuscript with the exact VIF range. The VIF values ranged from 1.341 to 1.397, all below the recommended threshold of 5, indicating that multicollinearity was not a serious concern in this study.

**Question 10**

5. Conceptual and Theoretical Comments

5.1 Theoretical Redundancy

The mediation logic between psychological safety and organizational support overlaps conceptually.

The manuscript needs:

Stronger theoretical differentiation between internal psychological resources vs external institutional resources.

Clear explanation why order is TPS → POS and not reversed.

Thanks. We agree that team psychological safety and perceived organizational support may appear conceptually related, and that the theoretical distinction and sequential rationale between these constructs should be more clearly articulated.

Following your suggestion, we revised the relevant section to reduce theoretical redundancy and strengthen the conceptual distinction between the two constructs. Specifically, team psychological safety was clarified as an interpersonal psychological climate within the team that reflects feelings of safety in communication and expression, whereas perceived organizational support was defined as nurses’ perceptions of institutional recognition and organizational resource support.

In addition, we further clarified the rationale for the proposed sequence of team psychological safety → perceived organizational support. In the revised manuscript, we explain that psychologically safe team environments may encourage nurses to communicate openly, express work-related needs, and actively participate in organizational affairs, which may subsequently enhance their perceptions of organizational support.

To improve clarity and avoid theoretical overlap, redundant theoretical explanations were streamlined and the serial mediation logic was presented more concisely in the revised manuscript.

**Question 11**

5.2 Cultural Interpretation Overreach

Section 4.1 attributes higher psychological safety to “China’s collectivist culture.”

This is speculative.

No cultural measures were included.

Recommendation:

Tone down cultural claims.

Present as possible explanation rather than conclusion.

Thanks. We agree that the cultural interpretation in the original manuscript may have been overstated, particularly because cultural variables were not directly measured in this study.

Following your suggestion, we revised the relevant discussion to substantially moderate the cultural claims. Specifically, the discussion now presents collectivist cultural characteristics as only one possible explanation for the findings rather than as a definitive conclusion. We also explicitly acknowledge that cultural factors were not empirically assessed in the present study and that this interpretation remains speculative.

These revisions have been incorporated into Section 4.1 of the revised manuscript to improve interpretive caution and theoretical rigor.

**Question 12**

6. Writing and Presentation Issues

6.1 Repetitive Discussion

Section 4.2 and 4.3 repeat theoretical explanations already presented in Introduction.

Recommendation:

Focus discussion on interpretation of effect sizes.

Compare mediation proportions with prior studies.

Reduce theoretical restatement.

Thanks. We agree that some parts of Sections 4.2 and 4.3 contained excessive theoretical repetition from the Introduction.

Following your suggestion, we substantially revised the Discussion section to reduce repeated theoretical explanations and place greater emphasis on interpreting the empirical findings. Specifically, we streamlined repetitive descriptions of SIP theory and social exchange theory, and instead focused on explaining the magnitude and practical meaning of the observed associations and indirect effects.

In addition, we strengthened comparisons between the present findings and previous studies, particularly regarding the mediating associations identified in this study. The revised discussion now places greater emphasis on the empirical contributions and practical implications of the findings rather than restating theoretical frameworks.

**Question 13**

6.2 Language Editing Required

Minor grammar and clarity issues appear throughout:

Examples:

“methods of sample size calculation” → “Sample size calculation”

“Not:” under Figure 2 → “Note:”

Inconsistent spacing around punctuation

Occasional awkward phrasing

Professional English editing is recommended.

Thanks. We carefully proofread and revised the manuscript to improve the overall language quality, grammatical accuracy, and clarity of expression.

Specifically, we corrected awkward wording, standardized terminology, revised minor grammatical issues, and improved punctuation and spacing consistency throughout the manuscript. In addition, several section titles and figure/table notes were refined for clearer academic English expression (e.g., revising “Sample size calculation method” to “Sample size calculation” and correcting “Not:” to “Note:”).

The manuscript has also undergone further language polishing to improve readability and overall presentation quality.

**Question 14**

7. Practical Implications Section

The three-dimensional framework:

Behavior activation

Psychological empowerment

Resource guarantee

This is strong but underdeveloped.

Recommendation:

Add 1–2 concrete implementation examples per dimension.

Provide actionable managerial guidance.

Thank you very much for this constructive suggestion and for recognizing the value of the proposed three-dimensional framework.

Following your recommendation, we further developed the practical implications section by adding concrete implementation examples for each dimension of the framework, including behavioral activation, psychological empowerment, and resource support. Specifically, we supplemented the revised manuscript with actionable nursing management strategies, such as encouraging nurses’ proactive participation in work redesign, fostering psychologically safe communication environments, and providing targeted organizational resources and innovation support during hospital relocation.

These additions were intended to improve the practical applicability and managerial relevance of the proposed framework.

**Question 15**

8. Minor Issues

Report exact p-values instead of p<0.001 where possible.

Clarify why 8-minute time limit was set.

Explain criteria for “unreasonable responses.”

Ensure all abbreviations defined once only.

Thanks. Following your recommendations, we carefully revised the manuscript to improve statistical reporting clarity and methodological transparency.

Specifically, exact p values were reported whenever available, while results displayed as “0.000” in the statistical output were appropriately revised to “p < 0.001.” In addition, we clarified the rationale for the 8-minute completion time threshold based on the pilot survey and average questionnaire completion time.

We also provided a clearer definition of “invalid/unreasonable responses,” including questionnaires with substantial missing data, obvious response regularity, contradictory responses, or excessively short completion times.

Finally, we carefully reviewed the entire manuscript to ensure that all abbreviations were defined only once and used consistently thereafter.

**Response to Reviewer 2,**

**Question 1**

The study title is okay. The abstract needs modifications. Make the sample's characteristics and setting clear, report important statistical indices (effect sizes, fit indices, and confidence intervals), outline the cross-sectional design's limitations with regard to causality, cut down on repetitive language, and sharpen the implications by connecting intervention strategies to results.

Thank you for this valuable comment. We have substantially revised the Abstract to improve its clarity, methodological transparency, and alignment with the journal style. Specifically, we clearly described the sample characteristics and study setting, added key statistical information including CFA model fit indices and mediation results, reduced repetitive expressions, and revised the wording to avoid causal overinterpretation associated with the cross-sectional design. In addition, we further clarified the practical implications by linking the findings to intervention strategies involving proactive work adjustment, psychological safety, and organizational support.

**Question 2**

Although conceptually sound, the introduction needs significant improvement. The majority of the literature review is descriptive, and it lacks a more thorough critical synthesis of earlier findings, particularly when it comes to contradictions in the available data. The suggested serial mediation sequence requires a more thorough explanation because the theoretical integration of COR, SIP, and SET seems disjointed. Despite the fact that a number of the cited studies are cross-sectional, causal language is overused. More attention must be paid to the research gap, novelty, and justification for concentrating on nurses in particular. There is an urgent need to include recent literature.

Specifically, we reduced overly descriptive literature summaries and strengthened the critical synthesis of previous findings, particularly regarding inconsistencies and limitations in existing studies. We also substantially revised the explanation of the proposed serial mediation pathway to improve the theoretical integration and reduce redundancy among COR theory, SIP theory, and social exchange theory. In the revised manuscript, the theoretical logic underlying the sequential association between team psychological safety and perceived organizational support is presented more coherently and concisely.

In addition, we carefully revised the manuscript to reduce causal language and replaced causal expressions with more appropriate associative terminology throughout the Introduction and related sections.

To strengthen the study rationale and novelty, we further clarified the research gaps, including the limited evidence from hospital relocation contexts, the lack of research focusing specifically on nurses, and the insufficient understanding of the sequential associations among interpersonal psychological resources and organizational support resources.

Finally, we updated the literature review by incorporating more recent studies to improve the timeliness and relevance of the theoretical background.

**Question 3**

Although the methods section is generally clear, there are a few things that could be done better. To address sampling bias and generalizability, explain the apparent discrepancy between convenience sampling and "random" recruitment and provide evidence for hospital selection. Describe the assumptions and justification for the adequacy of the SEM sample, the handling of missing data, and the exclusion criteria for "unreasonable responses". Give proof of validity (e.g., CFA indices) for the current sample's translated scales, particularly for the lower reliability coefficient (α = 0.728). Additionally, provide a clear explanation of any potential common-method biases and defend the 8-minute response limit.

Thanks. We have carefully revised the Methods section to improve methodological transparency and rigor. First, we clarified that the study adopted a convenience sampling strategy and revised the wording to avoid ambiguity regarding “random recruitment.” We also added a brief explanation for hospital selection. Second, we clarified that the final sample size met the recommended requirements for SEM analysis and further described the handling of missing data and invalid questionnaires. Specifically, questionnaires completed in less than 8 minutes, showing obvious response patterns, or containing substantial missing data were excluded. The 8-minute threshold was determined based on pilot testing and the estimated minimum time required to complete the questionnaires carefully. Third, to further support the validity of the translated scales, we added CFA results and supplementary reliability and validity indicators, including CR and AVE values. Although the Cronbach’s α coefficient for team psychological safety was relatively modest (α = 0.728), it remained within the acceptable range. Finally, we further clarified the assessment and limitations of common method bias in the revised manuscript.

**Question 4**

Although the results section is generally informative, there are a few things that could be done better. First, using Harman's single-factor test alone is not enough to evaluate common method bias; more robust techniques (e.g., CFA marker-variable approach) ought to be included. Second, considering the cross-sectional design, causal language in regression and SEM analyses is improper. Third, consistently report exact p values, effect sizes, standardized coefficients, and confidence intervals. Fourth, explain any differences between Table 1 (e) and significance statements. The g. professional title). Lastly, give full Table 5 values and explain how they were used.

Thanks. First, we acknowledge that Harman’s single-factor test alone may be insufficient for comprehensively assessing common method bias. Therefore, we further clarified the procedural controls adopted in this study and acknowledged the limitations of Harman’s test in the revised manuscript. Second, causal language throughout the Results and Discussion sections has been carefully revised and replaced with more appropriate associative terminology to better align with the cross-sectional design. Third, we revised the statistical reporting by adding more detailed information, including standardized coefficients, effect sizes, confidence intervals, and more precise p-values where appropriate. Fourth, we carefully rechecked Table 1 and corrected inconsistencies between the statistical results and narrative descriptions. Finally, Table 5 has been revised to provide complete mediation analysis results, including indirect effects and bootstrap confidence intervals, and additional explanations have been added accordingly.

**Question 5**

Theoretically, the discussion is pertinent, but it needs to be interpreted more precisely and critically. Instead of just restating the findings, the authors should expand the conversation by connecting the findings to more recent research in organizational psychology and nursing. There are still a number of conjectural explanations (e. The g. cultural collectivism, regional training disparities) and require cited empirical evidence. It is important to verify the accuracy of the confidence intervals for mediation effects because they seem to be reported inconsistently. The manuscript's rigor and scholarly quality would be significantly enhanced by placing more emphasis on alternative explanations, common method bias, causality limitations of cross-sectional design, and evidence-based practical implications.

Thank you for these insightful comments. We have substantially revised the Discussion section to improve its critical interpretation and academic rigor. Specifically, we reduced repetitive descriptions of the study results and placed greater emphasis on integrating the findings with recent research in organizational psychology and nursing management. In addition, several speculative interpretations, including cultural and regional explanations, were revised and presented more cautiously, with supporting empirical evidence added where appropriate. We also carefully rechecked the mediation effect confidence intervals to ensure consistency and accuracy throughout the manuscript. Furthermore, we strengthened the discussion of alternative explanations, common method bias, and the limitations of causal inference associated with the cross-sectional design. Finally, the practical implications section was refined to provide more evidence-based and actionable recommendations for nursing management practice.
