# Supplementary material for: Job crafting and innovative behavior among nurses: The serial mediating effect of team psychological safety and perceived organizational support
Source: PLoS One. 2026 Aug 10;21(8):e0352141. doi: 10.1371/journal.pone.0352141 (PMC13456283; doi:10.1371/journal.pone.0352141)
Supplement: Peer review history

# Peer review: Decision Letter 0

**DOI:** 10.1371/journal.pone.0352141.r001

**Authors:** Vinit Ramawat

5 May 2026

PONE-D-25-67625

Job Crafting and Innovative Behavior Among Nurses: The Serial Mediating Effect of Team Psychological Safety and Perceived Organizational Support

PLOS One

Dear Dr. Liu,

Thank you for submitting your manuscript to PLOS ONE. After careful consideration, we feel that it has merit but does not fully meet PLOS ONE’s publication criteria as it currently stands. Therefore, we invite you to submit a revised version of the manuscript that addresses the points raised during the review process.

Please submit your revised manuscript by Jun 19 2026 11:59PM. If you will need more time than this to complete your revisions, please reply to this message or contact the journal office at plosone@plos.org. When you're ready to submit your revision, log on to https://www.editorialmanager.com/pone/ and select the 'Submissions Needing Revision' folder to locate your manuscript file.

Please include the following items when submitting your revised manuscript:

- A letter that responds to each point raised by the academic editor and reviewer(s). You should upload this letter as a separate file labeled 'Response to Reviewers'.
- A marked-up copy of your manuscript that highlights changes made to the original version. You should upload this as a separate file labeled 'Revised Manuscript with Track Changes'.
- An unmarked version of your revised paper without tracked changes. You should upload this as a separate file labeled 'Manuscript'.

If you would like to make changes to your financial disclosure, please include your updated statement in your cover letter. Guidelines for resubmitting your figure files are available below the reviewer comments at the end of this letter.

If applicable, we recommend that you deposit your laboratory protocols in protocols.io to enhance the reproducibility of your results. Protocols.io assigns your protocol its own identifier (DOI) so that it can be cited independently in the future. For instructions see: https://journals.plos.org/plosone/s/submission-guidelines#loc-laboratory-protocols. Additionally, PLOS ONE offers an option for publishing peer-reviewed Lab Protocol articles, which describe protocols hosted on protocols.io. Read more information on sharing protocols at https://plos.org/protocols?utm_medium=editorial-email&utm_source=authorletters&utm_campaign=protocols.

As the corresponding author, your ORCID iD is verified in the submission system and will appear in the published article. PLOS supports the use of ORCID, and we encourage all coauthors to register for an ORCID iD and use it as well. Please encourage your coauthors to verify their ORCID iD within the submission system before final acceptance, as unverified ORCID iDs will not appear in the published article. *Only*  the individual author can complete the verification step; PLOS staff *cannot*  verify ORCID iDs on behalf of authors.

We look forward to receiving your revised manuscript.

Kind regards,

Vinit Kumar Ramawat, M.Sc Nursing, Ph.D Nursing

Guest Editor

PLOS One

Journal Requirements:

When submitting your revision, we need you to address these additional requirements.

1. Please ensure that your manuscript meets PLOS ONE's style requirements, including those for file naming. The PLOS ONE style templates can be found at

https://journals.plos.org/plosone/s/file?id=wjVg/PLOSOne_formatting_sample_main_body.pdf and https://journals.plos.org/plosone/s/file?id=ba62/PLOSOne_formatting_sample_title_authors_affiliations.pdf

2. Your ethics statement should only appear in the Methods section of your manuscript. If your ethics statement is written in any section besides the Methods, please delete it from any other section.

3. Thank you for stating the following financial disclosure:

The author(s) declare that financial support was received for the research and/or publication of this article. This study was funded by the Sichuan Hospital Management and Development Research Center, with the project number: SCYG2023-31.

Please state what role the funders took in the study. If the funders had no role, please state: "The funders had no role in study design, data collection and analysis, decision to publish, or preparation of the manuscript."

If this statement is not correct you must amend it as needed.

Please include this amended Role of Funder statement in your cover letter; we will change the online submission form on your behalf.

4. Please include captions for your Supporting Information files at the end of your manuscript, and update any in-text citations to match accordingly. Please see our Supporting Information guidelines for more information: http://journals.plos.org/plosone/s/supporting-information.

5. If the reviewer comments include a recommendation to cite specific previously published works, please review and evaluate these publications to determine whether they are relevant and should be cited. There is no requirement to cite these works unless the editor has indicated otherwise.

6. Please review your reference list to ensure that it is complete and correct. If you have cited papers that have been retracted, please include the rationale for doing so in the manuscript text, or remove these references and replace them with relevant current references. Any changes to the reference list should be mentioned in the rebuttal letter that accompanies your revised manuscript. If you need to cite a retracted article, indicate the article’s retracted status in the References list and also include a citation and full reference for the retraction notice.

[Note: HTML markup is below. Please do not edit.]

Reviewers' comments:

Reviewer's Responses to Questions

**Comments to the Author**

1. Is the manuscript technically sound, and do the data support the conclusions?

The manuscript must describe a technically sound piece of scientific research with data that supports the conclusions. Experiments must have been conducted rigorously, with appropriate controls, replication, and sample sizes. The conclusions must be drawn appropriately based on the data presented.

Reviewer #1: Partly

Reviewer #2: Yes

**********

2. Has the statistical analysis been performed appropriately and rigorously?

Reviewer #1: Yes

Reviewer #2: Yes

**********

3. Have the authors made all data underlying the findings in their manuscript fully available?

The PLOS Data policy requires authors to make all data underlying the findings described in their manuscript fully available without restriction, with rare exception (please refer to the Data Availability Statement in the manuscript PDF file). The data should be provided as part of the manuscript or its supporting information, or deposited to a public repository. For example, in addition to summary statistics, the data points behind means, medians and variance measures should be available. If there are restrictions on publicly sharing data—e.g. participant privacy or use of data from a third party—those must be specified.

Reviewer #1: No

Reviewer #2: Yes

**********

4. Is the manuscript presented in an intelligible fashion and written in standard English?

PLOS ONE does not copyedit accepted manuscripts, so the language in submitted articles must be clear, correct, and unambiguous. Any typographical or grammatical errors should be corrected at revision, so please note any specific errors here.

Reviewer #1: Yes

Reviewer #2: Yes

**********

5. Review Comments to the Author

Please use the space provided to explain your answers to the questions above. You may also include additional comments for the author, including concerns about dual publication, research ethics, or publication ethics. (Please upload your review as an attachment if it exceeds 20,000 characters)

Reviewer #1: 1. Overall Evaluation

This manuscript investigates the direct and serial mediating effects of team psychological safety and perceived organizational support on the relationship between job crafting and innovative behavior among nurses. The study is timely and relevant, particularly in healthcare systems where innovation and proactive behavior are increasingly required.

The topic aligns well with organizational psychology and nursing workforce research. The serial mediation model adds incremental value beyond simple direct-effect models.

However, several methodological, conceptual, and reporting issues must be addressed before the manuscript is suitable for publication.

2. Major Strengths

2.1 Clear Theoretical Framing

The integration of Conservation of Resources (COR) theory, Social Information Processing (SIP) theory, and Social Exchange Theory (SET) provides a solid conceptual backbone.

Hypotheses are logically structured and follow theory development.

2.2 Adequate Sample Size

Final sample size (n=449) exceeds the G*Power minimum requirement (n=204), increasing statistical reliability.

2.3 Appropriate Use of SEM

Structural equation modeling with bootstrapping (5,000 samples) is appropriate for mediation testing.

Model fit indices are acceptable (CFI=0.923, RMSEA=0.064).

3. Major Concerns (Require Revision)

3.1 Causality Claims in Cross-Sectional Design

The manuscript repeatedly uses causal language:

“predictive effect”

“causal relationships”

“transmission chain”

However, the study is cross-sectional. Causality cannot be inferred.

Recommendation:

Replace causal terminology with associative language (e.g., “associated with,” “related to”).

Revise Section 3.4 where the authors state they “verify causal relationships.”

Add a clearer limitation in Section 7 explicitly stating that mediation in cross-sectional data does not imply temporal causation.

3.2 Common Method Bias Assessment Is Insufficient

Harman’s single-factor test is widely criticized as inadequate.

Given:

All data are self-reported

Same-source measurement

Single time point

Risk of common method variance (CMV) is high.

Recommendation:

Conduct additional tests (e.g., CFA marker variable technique or unmeasured latent method factor).

At minimum, acknowledge Harman’s test limitations in the Discussion.

3.3 Convenience Sampling + “Random Recruitment” Inconsistency

Section 2.3 states:

“Convenience sampling method was used”

“500 nurses were randomly recruited”

This is contradictory.

Recommendation:

Clarify:

Was it convenience sampling at hospital level and random within hospitals?

Or purely convenience sampling?

Revise wording for methodological transparency.

3.4 Reliability of Team Psychological Safety Scale

Cronbach’s α = 0.728 in this study.

While acceptable, it is relatively low compared to original scale.

Recommendation:

Report item-total correlations.

Consider reporting composite reliability (CR) and AVE for SEM constructs.

Clarify whether CFA was conducted for measurement model.

3.5 Data Availability Statement Inconsistency

In the submission form:

“All relevant data are within the manuscript and its Supporting Information files.”

But in the manuscript:

“The raw data… will be made available by the authors…”

This is inconsistent with PLOS data policy.

Recommendation:

Ensure full compliance.

If data are not in repository, deposit in public repository before publication.

Revise manuscript to match submission declaration.

3.6 Hypothesis Numbering Errors

In Section 3.3:

Hypothesis 5 and Hypothesis 6 are mentioned

Earlier only 4 hypotheses are defined

This reflects structural inconsistency.

Recommendation:

Revise hypothesis numbering.

Ensure consistency between Introduction and Results.

4. Methodological Comments

4.1 Control Variables

Demographic variables significantly related to innovative behavior were identified (e.g., age, education, training).

However:

It is unclear whether these were controlled in SEM.

Recommendation:

Include significant demographics as covariates in the SEM model.

Clarify whether this was done.

4.2 Stepwise Regression

The manuscript uses stepwise regression.

Stepwise methods are statistically unstable and often discouraged.

Recommendation:

Replace with hierarchical regression based on theory.

Or remove regression section if SEM is primary analysis.

4.3 VIF Reporting

You report VIF < 5.

Provide:

Exact VIF range.

Clarify whether multicollinearity was tested before SEM.

5. Conceptual and Theoretical Comments

5.1 Theoretical Redundancy

The mediation logic between psychological safety and organizational support overlaps conceptually.

The manuscript needs:

Stronger theoretical differentiation between internal psychological resources vs external institutional resources.

Clear explanation why order is TPS → POS and not reversed.

5.2 Cultural Interpretation Overreach

Section 4.1 attributes higher psychological safety to “China’s collectivist culture.”

This is speculative.

No cultural measures were included.

Recommendation:

Tone down cultural claims.

Present as possible explanation rather than conclusion.

6. Writing and Presentation Issues

6.1 Repetitive Discussion

Section 4.2 and 4.3 repeat theoretical explanations already presented in Introduction.

Recommendation:

Focus discussion on interpretation of effect sizes.

Compare mediation proportions with prior studies.

Reduce theoretical restatement.

6.2 Language Editing Required

Minor grammar and clarity issues appear throughout:

Examples:

“methods of sample size calculation” → “Sample size calculation”

“Not:” under Figure 2 → “Note:”

Inconsistent spacing around punctuation

Occasional awkward phrasing

Professional English editing is recommended.

7. Practical Implications Section

The three-dimensional framework:

Behavior activation

Psychological empowerment

Resource guarantee

This is strong but underdeveloped.

Recommendation:

Add 1–2 concrete implementation examples per dimension.

Provide actionable managerial guidance.

8. Minor Issues

Report exact p-values instead of p<0.001 where possible.

Clarify why 8-minute time limit was set.

Explain criteria for “unreasonable responses.”

Ensure all abbreviations defined once only.

Reviewer #2: The study aims to support the improvement of nursing quality and nurses' professional development by offering intervention strategies to encourage the shift of nurses' innovative behaviours from passive compliance to proactive innovation. My observations are given below:

(1) The study title is okay. The abstract needs modifications. Make the sample's characteristics and setting clear, report important statistical indices (effect sizes, fit indices, and confidence intervals), outline the cross-sectional design's limitations with regard to causality, cut down on repetitive language, and sharpen the implications by connecting intervention strategies to results.

(2) Although conceptually sound, the introduction needs significant improvement. The majority of the literature review is descriptive, and it lacks a more thorough critical synthesis of earlier findings, particularly when it comes to contradictions in the available data. The suggested serial mediation sequence requires a more thorough explanation because the theoretical integration of COR, SIP, and SET seems disjointed. Despite the fact that a number of the cited studies are cross-sectional, causal language is overused. More attention must be paid to the research gap, novelty, and justification for concentrating on nurses in particular. There is an urgent need to include recent literature.

(3) Although the methods section is generally clear, there are a few things that could be done better. To address sampling bias and generalizability, explain the apparent discrepancy between convenience sampling and "random" recruitment and provide evidence for hospital selection. Describe the assumptions and justification for the adequacy of the SEM sample, the handling of missing data, and the exclusion criteria for "unreasonable responses". Give proof of validity (e.g., CFA indices) for the current sample's translated scales, particularly for the lower reliability coefficient (α = 0.728). Additionally, provide a clear explanation of any potential common-method biases and defend the 8-minute response limit.

(4) Although the results section is generally informative, there are a few things that could be done better. First, using Harman's single-factor test alone is not enough to evaluate common method bias; more robust techniques (e.g., CFA marker-variable approach) ought to be included. Second, considering the cross-sectional design, causal language in regression and SEM analyses is improper. Third, consistently report exact p values, effect sizes, standardized coefficients, and confidence intervals. Fourth, explain any differences between Table 1 (e) and significance statements. The g. professional title). Lastly, give full Table 5 values and explain how they were used.

(5) Theoretically, the discussion is pertinent, but it needs to be interpreted more precisely and critically. Instead of just restating the findings, the authors should expand the conversation by connecting the findings to more recent research in organizational psychology and nursing. There are still a number of conjectural explanations (e. The g. cultural collectivism, regional training disparities) and require cited empirical evidence. It is important to verify the accuracy of the confidence intervals for mediation effects because they seem to be reported inconsistently. The manuscript's rigor and scholarly quality would be significantly enhanced by placing more emphasis on alternative explanations, common method bias, causality limitations of cross-sectional design, and evidence-based practical implications.

**********

6. PLOS authors have the option to publish the peer review history of their article (what does this mean?). If published, this will include your full peer review and any attached files.

If you choose “no”, your identity will remain anonymous but your review may still be made public.

**Do you want your identity to be public for this peer review?**  For information about this choice, including consent withdrawal, please see our Privacy Policy.

Reviewer #1: **Yes:**  Ebtsam Aly Abou Hashish

Reviewer #2: **Yes:**  Gyanesh Kumar Tiwari

**********

[NOTE: If reviewer comments were submitted as an attachment file, they will be attached to this email and accessible via the submission site. Please log into your account, locate the manuscript record, and check for the action link "View Attachments". If this link does not appear, there are no attachment files.]

To ensure your figures meet our technical requirements, please review our figure guidelines: https://journals.plos.org/plosone/s/figures

You may also use PLOS’s free figure tool, NAAS, to help you prepare publication quality figures: https://journals.plos.org/plosone/s/figures#loc-tools-for-figure-preparation.

NAAS will assess whether your figures meet our technical requirements by comparing each figure against our figure specifications.

# Author comment: Author response to Decision Letter 1

**DOI:** 10.1371/journal.pone.0352141.r002

26 May 2026

Dear Editor and Reviewers,

We sincerely thank the Editor and Reviewers for the careful evaluation of our manuscript and for the constructive comments and valuable suggestions. We have carefully revised the manuscript according to all comments and believe that these revisions have substantially improved the clarity, rigor, and overall quality of the manuscript. All changes have been highlighted in the revised version, and detailed responses to each comment are provided below.

Response to Reviewer 1,

Question 1

3.1 Causality Claims in Cross-Sectional Design

The manuscript repeatedly uses causal language:

“predictive effect”

“causal relationships”

“transmission chain”

However, the study is cross-sectional. Causality cannot be inferred.

Recommendation:

Replace causal terminology with associative language (e.g., “associated with,” “related to”).

Revise Section 3.4 where the authors state they “verify causal relationships.”

Add a clearer limitation in Section 7 explicitly stating that mediation in cross-sectional data does not imply temporal causation.

Thank you very much for this insightful comment. We fully agree that causal inferences cannot be established based on a cross-sectional design. Following your suggestion, we carefully revised the manuscript to avoid causal language throughout the text.

Specifically, terms such as “predictive effect,” “causal relationship,” and “transmission chain” were replaced with more appropriate associative expressions, including “association,” “relationship,” and “serial mediating association.” In Section 3.4, the statement regarding “verification of causal relationships” has been revised to reflect statistical associations rather than causality. In addition, we strengthened the limitations section by explicitly stating that mediation effects identified from cross-sectional data do not imply temporal or causal relationships among variables.

These revisions have been incorporated throughout the manuscript to ensure conceptual and methodological accuracy.

Question 2

3.2 Common Method Bias Assessment Is Insufficient

Harman’s single-factor test is widely criticized as inadequate.

Given:

All data are self-reported

Same-source measurement

Single time point

Risk of common method variance (CMV) is high.

Recommendation:

Conduct additional tests (e.g., CFA marker variable technique or unmeasured latent method factor).

At minimum, acknowledge Harman’s test limitations in the Discussion.

Following your suggestion, we have revised the manuscript in several ways. First, we moderated the interpretation of the Harman’s single-factor test results in Section 3.1. Specifically, instead of stating that “there is no significant common method bias,” we now indicate that the findings only suggest that CMV may not seriously threaten the study results, while acknowledging the recognized limitations of Harman’s test.

Second, we added a clearer discussion in the limitations section to acknowledge the potential risk of CMV associated with the cross-sectional self-report design. We also noted that Harman’s single-factor test cannot completely rule out CMV effects and suggested that future studies adopt more rigorous approaches, such as longitudinal designs, multi-source data collection, or latent method factor techniques.

In addition, we clarified the procedural remedies used to reduce CMV risk, including anonymous participation, voluntary responses, confidentiality assurances, and standardized questionnaire instructions.

These revisions have been incorporated into the revised manuscript accordingly.

Question 3

3.3 Convenience Sampling + “Random Recruitment” Inconsistency

Section 2.3 states:

“Convenience sampling method was used”

“500 nurses were randomly recruited”

This is contradictory.

Recommendation:

Clarify:

Was it convenience sampling at hospital level and random within hospitals?

Or purely convenience sampling?

Revise wording for methodological transparency.

Thanks. We agree that the description of the sampling procedure in the original manuscript was unclear and could lead to confusion between convenience sampling and random sampling.

In the revised manuscript, we clarified the sampling strategy by removing the term “randomly recruited.” The study actually adopted a convenience sampling approach, in which eligible nurses from three Grade A tertiary general hospitals in western China were invited to voluntarily participate in the survey.

The relevant description in Section 2.3 has been revised accordingly to improve methodological transparency and accuracy.

Question 4

3.4 Reliability of Team Psychological Safety Scale

Cronbach’s α = 0.728 in this study.

While acceptable, it is relatively low compared to original scale.

Recommendation:

Report item-total correlations.

Consider reporting composite reliability (CR) and AVE for SEM constructs.

Clarify whether CFA was conducted for measurement model.

Thank you very much for this constructive comment. Following your suggestion, we further evaluated the measurement properties of the constructs by conducting CFA and calculating CR and AVE.

The CFA results demonstrated an acceptable model fit (χ²/df = 2.822, RMSEA = 0.064, CFI = 0.923, TLI = 0.908, IFI = 0.924). For the Team Psychological Safety Scale, the Cronbach’s α coefficient was 0.728 and the CR value was 0.736, indicating acceptable internal consistency reliability. Although the AVE value was below the recommended threshold of 0.50, the CR exceeded 0.70, which still suggests acceptable convergent validity according to the criterion proposed by Fornell and Larcker.

In addition, item-total correlations and measurement model information have been supplemented in the revised manuscript to improve methodological transparency and rigor.

Question 5

3.5 Data Availability Statement Inconsistency

In the submission form:

“All relevant data are within the manuscript and its Supporting Information files.”

But in the manuscript:

“The raw data… will be made available by the authors…”

This is inconsistent with PLOS data policy.

Recommendation:

Ensure full compliance.

If data are not in repository, deposit in public repository before publication.

Revise manuscript to match submission declaration.

Thank you very much for pointing out the inconsistency in the Data Availability Statement.

Following the journal’s data-sharing policy and your suggestion, we have revised the manuscript to ensure full compliance with PLOS data availability requirements. Specifically, the de-identified raw dataset has now been deposited in a public repository (Figshare), and the corresponding DOI link has been added to the revised manuscript.

The previous statement indicating that the data would be available “upon reasonable request” has been removed to avoid inconsistency with the submission information.

The revised Data Availability Statement is as follows:

“The de-identified datasets generated and analyzed during the current study are publicly available in the Figshare repository: https://doi.org/10.6084/m9.figshare.32408385”

Question 6

3.6 Hypothesis Numbering Errors

In Section 3.3:

Hypothesis 5 and Hypothesis 6 are mentioned

Earlier only 4 hypotheses are defined

This reflects structural inconsistency.

Recommendation:

Revise hypothesis numbering.

Ensure consistency between Introduction and Results.

Thank you very much for this careful observation. We apologize for the inconsistency in the hypothesis numbering between the Introduction and Results sections.

Following your suggestion, we carefully checked and revised the hypothesis numbering throughout the manuscript to ensure consistency and structural accuracy. The incorrect references to “Hypothesis 5” and “Hypothesis 6” in Section 3.3 have been corrected to match the four hypotheses originally proposed in the Introduction.

All related sections, tables, and figure descriptions have also been reviewed and revised accordingly.

Question 7

4. Methodological Comments

4.1 Control Variables

Demographic variables significantly related to innovative behavior were identified (e.g., age, education, training).

However:

It is unclear whether these were controlled in SEM.

Recommendation:

Include significant demographics as covariates in the SEM model.

Clarify whether this was done.

Thanks. We agree that demographic variables significantly associated with innovative behavior may potentially influence the relationships examined in the SEM model.

In the present study, the primary focus was on the associations among job crafting, team psychological safety, perceived organizational support, and innovative behavior. Demographic variables were examined in the preliminary analyses; however, they were not included as covariates in the final SEM model. We acknowledge that this may limit the ability to fully account for potential confounding effects.

Following your suggestion, we have clarified this issue in the revised manuscript and added it to the limitations section.

Question 8

4.2 Stepwise Regression

The manuscript uses stepwise regression.

Stepwise methods are statistically unstable and often discouraged.

Recommendation:

Replace with hierarchical regression based on theory.

Or remove regression section if SEM is primary analysis.

Thanks. We agree that stepwise regression methods may have limitations in terms of statistical stability and theoretical interpretability.

Considering that structural equation modeling (SEM) was the primary analytical approach of this study, we carefully reconsidered the necessity of the stepwise regression analysis. Following your suggestion, we removed the stepwise regression results from the revised manuscript and retained SEM as the main analytical framework for examining the relationships among variables.

The manuscript has been revised accordingly to improve methodological rigor and consistency.

Question 9

4.3 VIF Reporting

You report VIF < 5.

Provide:

Exact VIF range.

Clarify whether multicollinearity was tested before SEM.

Thank you very much for this helpful suggestion. We agree that more detailed reporting of multicollinearity diagnostics would improve the transparency of the analysis.

Following your recommendation, we clarified in the revised manuscript that multicollinearity diagnostics were conducted prior to the SEM analysis. Specifically, VIF values were calculated to assess potential multicollinearity among the study variables.

In addition, we supplemented the manuscript with the exact VIF range. The VIF values ranged from 1.341 to 1.397, all below the recommended threshold of 5, indicating that multicollinearity was not a serious concern in this study.

Question 10

5. Conceptual and Theoretical Comments

5.1 Theoretical Redundancy

The mediation logic between psychological safety and organizational support overlaps conceptually.

The manuscript needs:

Stronger theoretical differentiation between internal psychological resources vs external institutional resources.

Clear explanation why order is TPS → POS and not reversed.

Thanks. We agree that team psychological safety and perceived organizational support may appear conceptually related, and that the theoretical distinction and sequential rationale between these constructs should be more clearly articulated.

Following your suggestion, we revised the relevant section to reduce theoretical redundancy and strengthen the conceptual distinction between the two constructs. Specifically, team psychological safety was clarified as an interpersonal psychological climate within the team that reflects feelings of safety in communication and expression, whereas perceived organizational support was defined as nurses’ perceptions of institutional recognition and organizational resource support.

In addition, we further clarified the rationale for the proposed sequence of team psychological safety → perceived organizational support. In the revised manuscript, we explain that psychologically safe team environments may encourage nurses to communicate openly, express work-related needs, and actively participate in organizational affairs, which may subsequently enhance their perceptions of organizational support.

To improve clarity and avoid theoretical overlap, redundant theoretical explanations were streamlined and the serial mediation logic was presented more concisely in the revised manuscript.

Question 11

5.2 Cultural Interpretation Overreach

Section 4.1 attributes higher psychological safety to “China’s collectivist culture.”

This is speculative.

No cultural measures were included.

Recommendation:

Tone down cultural claims.

Present as possible explanation rather than conclusion.

Thanks. We agree that the cultural interpretation in the original manuscript may have been overstated, particularly because cultural variables were not directly measured in this study.

Following your suggestion, we revised the relevant discussion to substantially moderate the cultural claims. Specifically, the discussion now presents collectivist cultural characteristics as only one possible explanation for the findings rather than as a definitive conclusion. We also explicitly acknowledge that cultural factors were not empirically assessed in the present study and that this interpretation remains speculative.

These revisions have been incorporated into Section 4.1 of the revised manuscript to improve interpretive caution and theoretical rigor.

Question 12

6. Writing and Presentation Issues

6.1 Repetitive Discussion

Section 4.2 and 4.3 repeat theoretical explanations already presented in Introduction.

Recommendation:

Focus discussion on interpretation of effect sizes.

Compare mediation proportions with prior studies.

Reduce theoretical restatement.

Thanks. We agree that some parts of Sections 4.2 and 4.3 contained excessive theoretical repetition from the Introduction.

Following your suggestion, we substantially revised the Discussion section to reduce repeated theoretical explanations and place greater emphasis on interpreting the empirical findings. Specifically, we streamlined repetitive descriptions of SIP theory and social exchange theory, and instead focused on explaining the magnitude and practical meaning of the observed associations and indirect effects.

In addition, we strengthened comparisons between the present findings and previous studies, particularly regarding the mediating associations identified in this study. The revised discussion now places greater emphasis on the empirical contributions and practical implications of the findings rather than restating theoretical frameworks.

Question 13

6.2 Language Editing Required

Minor grammar and clarity issues appear throughout:

Examples:

“methods of sample size calculation” → “Sample size calculation”

“Not:” under Figure 2 → “Note:”

Inconsistent spacing around punctuation

Occasional awkward phrasing

Professional English editing is recommended.

Thanks. We carefully proofread and revised the manuscript to improve the overall language quality, grammatical accuracy, and clarity of expression.

Specifically, we corrected awkward wording, standardized terminology, revised minor grammatical issues, and improved punctuation and spacing consistency throughout the manuscript. In addition, several section titles and figure/table notes were refined for clearer academic English expression (e.g., revising “Sample size calculation method” to “Sample size calculation” and correcting “Not:” to “Note:”).

The manuscript has also undergone further language polishing to improve readability and overall presentation quality.

Question 14

7. Practical Implications Section

The three-dimensional framework:

Behavior activation

Psychological empowerment

Resource guarantee

This is strong but underdeveloped.

Recommendation:

Add 1–2 concrete implementation examples per dimension.

Provide actionable managerial guidance.

Thank you very much for this constructive suggestion and for recognizing the value of the proposed three-dimensional framework.

Following your recommendation, we further developed the practical implications section by adding concrete implementation examples for each dimens

Attachment Submitted filename: Response to Reviewers.doc

# Editor report: Decision Letter 1

**DOI:** 10.1371/journal.pone.0352141.r003

**Authors:** Vinit Ramawat

7 Jun 2026

<p>Job Crafting and Innovative Behavior Among Nurses: The Serial Mediating Effect of Team Psychological Safety and Perceived Organizational Support

PONE-D-25-67625R1

Dear Dr. Liu,

We’re pleased to inform you that your manuscript has been judged scientifically suitable for publication and will be formally accepted for publication once it meets all outstanding technical requirements.

Within one week, you’ll receive an e-mail detailing the required amendments. When these have been addressed, you’ll receive a formal acceptance letter and your manuscript will be scheduled for publication.

An invoice will be generated when your article is formally accepted. Please note, if your institution has a publishing partnership with PLOS and your article meets the relevant criteria, all or part of your publication costs will be covered. Please make sure your user information is up-to-date by logging into Editorial Manager at Editorial Manager® and clicking the ‘Update My Information' link at the top of the page. For questions related to billing, please contact billing support.

If your institution or institutions have a press office, please notify them about your upcoming paper to help maximize its impact. If they’ll be preparing press materials, please inform our press team as soon as possible -- no later than 48 hours after receiving the formal acceptance. Your manuscript will remain under strict press embargo until 2 pm Eastern Time on the date of publication. For more information, please contact onepress@plos.org.

Kind regards,

Vinit Kumar Ramawat, M.Sc Nursing, Ph.D Nursing

Guest Editor

PLOS One

Additional Editor Comments (optional):

Reviewers' comments:

# Editor report: Acceptance letter

**DOI:** 10.1371/journal.pone.0352141.r004

**Authors:** Vinit Ramawat

PONE-D-25-67625R1

PLOS One

Dear Dr. Liu,

I'm pleased to inform you that your manuscript has been deemed suitable for publication in PLOS One. Congratulations! Your manuscript is now being handed over to our production team.

At this stage, our production department will prepare your paper for publication. This includes ensuring the following:

* All references, tables, and figures are properly cited

* All relevant supporting information is included in the manuscript submission,

* There are no issues that prevent the paper from being properly typeset

You will receive further instructions from the production team, including instructions on how to review your proof when it is ready. Please keep in mind that we are working through a large volume of accepted articles, so please give us a few days to review your paper and let you know the next and final steps.

Lastly, if your institution or institutions have a press office, please let them know about your upcoming paper now to help maximize its impact. If they'll be preparing press materials, please inform our press team within the next 48 hours. Your manuscript will remain under strict press embargo until 2 pm Eastern Time on the date of publication. For more information, please contact onepress@plos.org.

You will receive an invoice from PLOS for your publication fee after your manuscript has reached the completed accept phase. If you receive an email requesting payment before acceptance or for any other service, this may be a phishing scheme. Learn how to identify phishing emails and protect your accounts at https://explore.plos.org/phishing.

If we can help with anything else, please email us at customercare@plos.org.

Thank you for submitting your work to PLOS ONE and supporting open access.

Kind regards,

PLOS ONE Editorial Office Staff

on behalf of

Dr. Vinit Kumar Ramawat

Guest Editor

PLOS One
